# Successful management of vulvar proximal-type epithelioid sarcoma in pregnancy

**DOI:** 10.1016/j.gore.2022.100933

**Published:** 2022-01-21

**Authors:** Yuji Orita, Masaki Kamio, Akio Tokudome, Ikumi Kitazono, Fumino Ichihara, Hiroaki Kobayashi

**Affiliations:** a Department of Obstetrics and Gynecology Faculty of Medicine, Kagoshima University, Japan; b Department of Obstetrics and Gynecology, Kanoya Medical Center, Japan; c Department of Pathology, Kagoshima University Graduate School of Medical and Dental Sciences, Japan

**Keywords:** Pregnancy, Proximal-type epithelioid sarcoma, Vulvar, Long outcome

## Abstract

Vulvar proximal-type epithelioid sarcoma during pregnancy is extremely rare; only two reports are available to date. Herein, we describe a 36-year-old woman who presented with a pigeon-egg-sized solid mass with cystic component on the left labia majora at 18 weeks of gestation. The patient underwent tumor resection at 23 weeks of gestation and was diagnosed with epithelioid sarcoma, proximal-type. At 29 weeks of gestation, elective cesarean section, radical local resection of the vulva and vagina, and inguinal lymphadenectomy were performed. After surgery, she underwent six courses of adjuvant chemotherapy (doxorubicin 60 mg/m^2^ and cisplatin 50 mg/m^2^) every four weeks. The patient and her baby survived with neither recurrence nor complications until 5 years. Aggressive management for proximal-type epithelioid sarcoma, such as early termination of pregnancy and operation, can improve maternal outcomes.

## Highlights

• Vulvar proximal-type epithelioid sarcoma during pregnancy is extremely rare.
• Immediate and agressive treatment is essential even in pregnancy, and vulvectomy during pregnancy is considered safe.
• Further reserch is required to discuss about the effectiveness of adjuvant therapy.

## 1 Introduction

Malignancies of the vulva are rare (Altundag et al., 2004). Particularly, proximal-type epithelioid sarcoma (PES) of the vulva is extremely rare; it accounts for only 1–3% of vulvar malignancies (Rodrigues et al., 2015, Tjalma et al., 1999). However, clear management guidelines and optimal therapies for this malignancy have not been established. Epithelioid sarcoma (ES) was first reported in 1970. It is characterized by a painless nodule or mass with a poor prognosis (Enzinger, 1970). In an article describing 37 cases, the recurrence rate during follow-up was 43.2%, with a mean disease survival time of 17.2 months. Overall, 27.0% died of the disease (Han et al., 2016). When ES occurs in Bartholin’s gland, it can be misdiagnosed as benign tumor and lead to tumor advancement (Matsuo et al., 2009, Rodrigues et al., 2015, Tjalma et al., 1999). Early diagnosis and appropriate treatment are essential to improve prognosis and survival in patients with ES. However, there is no established management criteria, especially in pregnancy, owing to its rarity. Although a previous report presented two cases of PES in pregnancy (Moore et al., 2002, Rai et al., 2009), no successful management in pregnancy was achieved. This report is the first to describe successful management of PES during pregnancy.

## 2 Case report

A 36-year-old healthy woman, gravida 2 para 1 had presented to another hospital with a pigeon-egg-sized cystic tumor on the left labia majora at 18 weeks of gestation in 2016. She had noticed a soybean-sized elastic mass on the left vulva six months previously. The tumor gradually grew to a pigeon-egg size and was diagnosed as Bartholin’s gland cyst. A 5-cm asymptomatic smooth elastic mass was found on the left labia majora and did not extend to the vagina. The tumor was resected at 23 weeks of gestation at Kanoya Medical Center. Moreover, a histopathological diagnosis of PES, proximal-type, was made. The tumor was a solid mass with hemorrhagic cyst, 50x30x10 mm in size. Microscopically, the tumor consisted of a multinodular proliferation of epithelioid or polygonal tumor cells with hemorrhage and necrosis within the nodules (Fig. 1a). Immunohistochemical analysis showed that the tumor cells were positive for epithelial marker: cytokeratin(AE1/AE3)and mesenchymal marker: vimentin and partially positive for CD34. The tumor cells lacked nuclear expression of INI-1 (Fig. 1b) and were negative for S-100, HMB-45, MDM-2, CDK4, and desmin. There was no metastasis. However, residual tumor was suspected on pelvic magnetic resonance imaging (MRI) and whole-body contrast-enhanced computed tomography (CT) after the first operation. She visited our institution for advanced therapy. Following antenatal steroid administration (betamethasone 12 mg for 2 days), elective cesarean section, radical local resection of the vulva and vagina, and left inguinal lymphadenectomy were performed at 29 weeks and 6 days of gestation. The resected residual tumor was 5 mm in size. However, the surgical margin was positive, close to the external urethral orifice. No lymph node metastasis was observed. After the second operation, she was diagnosed with stage Ⅱ vulvar PES according to the The International Federation of Gynecology and Obstetrics staging system (FIGO 2008). The male newborn weighed 1,508 g (appropriate for gestational age), and the Apgar score was 7/9 (at 1 min/5 min). He was intubated for respiratory distress syndrome for 1 day and subsequently discharged uneventfully. Postoperatively, the patient experienced complications, including abundant lymphatic leakage and surgical site infection of inguinal lesions, and required one month for complete recovery. Inguinal lymphatic leakage volume amounted to over 500 mL per day. One month after the second operation, adjuvant chemotherapy (doxorubicin 60 mg/m^2^ and cisplatin 50 mg/m^2^; six courses) was commenced every four weeks. The patient and her baby survived with neither recurrence nor complications until 5 years.

**Fig. 1a:**
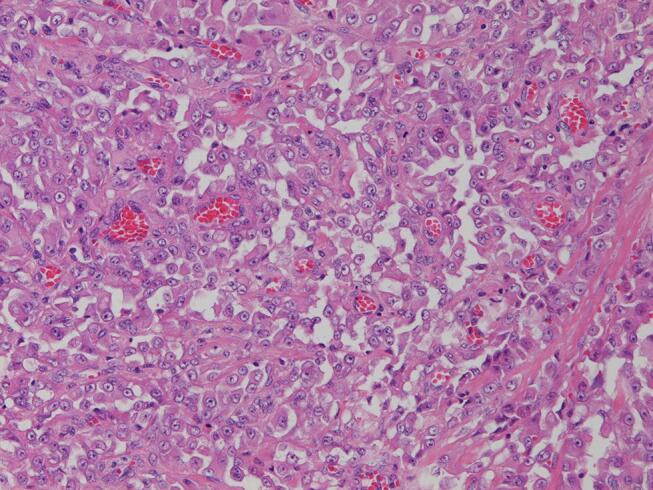
HE stain (×200). The tumor cells are epithelioid with abundant eosinophilic cytoplasm and round nuclei with prominent nucleoli.

**Fig. 1b:**
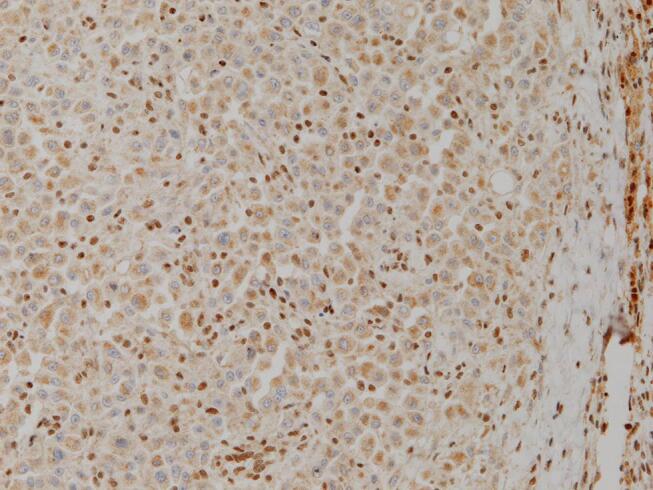
Immunohistochemical staining of INI-1 (×200). Loss of INI-1 expression in nuclear of tumor cells; background inflammatory cells positive for INI-1.

## 3 Discussion

Cancer during pregnancy is relatively rare; approximately 0.1–0.2% of women have cancer during pregnancy (Amant et al., 2014). Particularly, vulvar PES during pregnancy is extremely rare (Matsuo et al., 2014). Vulvar sarcomas usually occur on the left labia majora (Matsuo et al., 2009), as was seen in this case. Therefore, approximately one-third of these cases are initially misdiagnosed as a Bartholin’s gland cyst or abscess (Matsuo et al., 2009), as observed in this case. Misdiagnosis often leads to tumor progression and worse prognosis due to delayed treatment (Altundag et al., 2004, Matsuo et al., 2009, Rodrigues et al., 2015, Tjalma et al., 1999). The mean age of PES occurrence is approximately 27.8–35 years, similar to that in patients with other sarcomas (Altundag et al., 2004, Matsuo et al., 2009, Rodrigues et al., 2015, Tjalma et al., 1999). Obstetricians widely need to know that when women in reproductive age present with left-sided vulvar tumor, vulvar sarcomas must be ruled out.

PES is an extremely rare soft-tissue tumor of mesenchymal origin (Altundag et al., 2004, Argenta et al., 2007, Rodrigues et al., 2015). It presents aggressively, characterized by rapid expansion, frequent local recurrence with negative margins, and early metastasis after onset with poor prognosis (Argenta et al., 2007, Rodrigues et al., 2015). ES was was first reported in 1970 (Enzinger, 1970), which is malignant mesenchymal tumor of uncertain differentiation according to the WHO classification. This tumor is characterized by proliferation of epithelioid tumor cells that exhibit multinodular or necrotic granulomatous growth pattern. Furthermore, there are two subtypes of ES: classic (distal or conventional) type and proximal-type. Classic ES usually occurs in the distal extremities. Proximal-type epithelioid sarcoma (PES) is an agressive subtype which was first reported by Gulliou in 1997 (Gulliou et al., 1997). PES mostly occurs in the proximal limbs, pelvis, perineum, and genital tract. Tumor size >5 cm, high-grade tumors (grade 3 and 4), tumor necrosis >50%, vascular invasion, proximal-type, presence of lymph node metastasis have been reported to be prognostic and predictive factors for ES (Czarneckaet al., 2020). Unlike classic ES, PES is microscopically characterized by predominantly large-cell, epithelioid cytomorphology, marked cytologic atypia, frequent occurence of rhabdoid features, and lack of granulomatous growth pattern (Gulliou et al., 1997). PES originates from the superficial or deep tissue of the vulva and manifests as single or multiple nodules containing focal areas of necrosis and hemorrhage (Altundag et al., 2004, Rodrigues et al., 2015, Tjalma et al., 1999). Immunohistochemical staining is useful for microscopic diagnosis of ES, by which lack of staining for INI-1 protein is a characteristic finding (Kim et al., 2012). In the present case, these features supported the pathological diagnosis of PES.

Only two vulvar PES cases during pregnancy have been reported in the English literature (Moore et al., 2002, Rai et al., 2009). One case involved a 29-year-old woman at 36 weeks of gestation with multiple lung metastases. She underwent radical hemivulvectomy six weeks after delivery followed by chemotherapy (doxorubicin 50 mg/m^2^ and ifosfamide 1.5 g/m^2^). Despite these therapies, she died 5 months after delivery (Moore et al., 2002). Another case involved a 17-year-old African American in the first trimester with inguinal node metastasis. She had not received additional therapy at 16 weeks of gestation. However, long-term outcome in that case was not described (Rai et al., 2009). In our case, we performed tumor resection at 23 weeks of gestation and established a diagnosis. Elective cesarean section, radical local resection of the vulva and vagina, and left inguinal lymphadenectomy were performed at 29 weeks of gestation. In vulvar cancer with clinically negative nodes diagnosed before 36 weeks of gestation, radical vulvectomy with lymphadenectomy is recommended (Amant et al., 2014). In cases with clinically positive nodes, immediate termination of pregnancy and surgical treatment are required (Amant et al., 2014). Additionally, planned cesarean section is the preferred method in cases of confirmed malignancy in view of potential local recurrence and distant metastasis (Amant et al., 2014). Regarding PES, immediate treatment is essential owing to its unfavorable prognosis. Vulvectomy during pregnancy is considered safe (Matsuo et al., 2014). Genital vascularization is markedly increased during pregnancy, and surgery tends to result in higher operative blood loss. In this case, the total blood loss, including volume lost during the cesarean section, was 1,250 mL. After the operation, abundant lymph fluid was drained from the resected left inguinal lymph nodes. In addition, surgical site infection took one month to heal. Whether adjuvant therapies such as radiotherapy and chemotherapy improve outcomes remains unclear even in these days (Czarnecka et al., 2020). However, most advanced cases may be challenging to treat with adjuvant therapy. In our case, the diagnosis made at an early-stage before metastasis. However, for residual tumor, adjuvant chemotherapy (doxorubicin 60 mg/m^2^ and cisplatin 50 mg/m^2^; six courses) was administered every 4 weeks. In ES cases, single doxorubicin or doworubicin with ifosfamide (or with other cytotoxic drugs or single agents) is commonly choosen (Czarnecka et al., 2020). However, we selected doxorubicin and cisplatin due to unfavorable outcomes of previously reported case (Moore et al., 2002). Recently, it has been reported that anthracycline-based and gemcitabine-based regimens showed a similar response rate. However, the value of pazopanib was low response rate for ES (Frezza et al., 2018). Since, we cannnot confirm the effectiveness of cisplatin. *SMARCB1/INI1 gene* located on chromosome 22q11.2 acts as a tumor suppressor gene and play a crucial role in ES (Hornick et al., 2009). Gene therapy targeting the genetic pathway in tumorgenesis of this aggressive neoplasm may provide a better prognosis. However, such gene therapy is not common in 2021. Futher research is required.

In vulvar PES cases, it is often misdiagnosed initially as Bartholin’s gland cyst. During pregnancy, misdiagnosis might happen more easily due to rarerity of vulvar PES and concerns over possible maternal-fetal adverse effects of surgical treatment. However, early-stage diagnosis and aggressive approaches such as early termination of pregnancy, surgical resection, and adjuvant therapy can improve maternal outcomes. Moreover, accurate diagnosis and management according to gestational age are important.

### CRediT authorship contribution statement

**Yuji Orita:** Conceptualization, Writing – original draft. **Masaki Kamio:** Project administration, Writing – review & editing. **Akio Tokudome:** Resources, Writing – review & editing. **Ikumi Kitazono:** Resources, Writing – review & editing. **Fumino Ichihara:** Resources, Writing – review & editing. **Hiroaki Kobayashi:** Supervision.

## Declaration of Competing Interest

The authors declare that they have no known competing financial interests or personal relationships that could have appeared to influence the work reported in this paper.
